# Semen banking: consideration on viral contamination in the era of new emerging viral infection

**Published:** 2011

**Authors:** Viroj Wiwanitkit

**Affiliations:** Wiwanitkit House, Bangkhae, Bangkok, Thailand.

To construct a semen bank, the collection of donated semen has to be done and an important concern is the safety of collected semen. The contamination is a big problem. Basically, the infectious pathogens can exist within donated semen, hence, a good donor screening is very important. Although viruses have an indirect role in sperm quality, but the evidence in banked semen is presently lack. This does not mean that there is no viral contamination but it might imply the inadequate concern on this issue. Contaminated semen usually means poor quality and hazardous to the recipient. The contamination of the virus in banked semen is a common problem in animal semen banking (1). The safety and transmission of each problematic virus is widely studied and well clarified in animal semen banking (2). However, this issue is not widely concerned in human semen banking. For sure, this case is an actual direct contamination and this cannot be detected if there is no specific screening in the banking process. The scenario of important new emerging viral infections will be specifically detailed in this report. West Nile virus is an emerging problematic viral infection that can cause a deadly clinical disorder. Basically, West Nile virus is classified as an arbovirus that is mainly transmitted by mosquito. However, the uncommon modes of transmissions such as transfusion related transmission are reported (3). The contamination of West Nile virus in semen is an important question in andrology. There is no evidence indicating for the presence of West Nile virus in the semen of the patients. However, American Society for Reproductive Medicine/Society for Assisted Reproductive Technology recommended that practitioners defer gamete donors who have confirmed or suspected West Nile virus infections (4). 

SARS is another deadly emerging viral infection. The new coronavirus infection is transmitted via respiratory route. The serious symptom due to this infection leads to death in almost all cases and brings a great concern to medical scientists around the world. The contamination of SARS in semen is an interesting topic. The possible transmission of SARS virus via germ line is an important question to be investigated in reproductive medicine (5). Luckily, till present, there is no evidence of SARS contamination in semen. Generally, influenza virus is a respiratory virus that causes respiratory tract infection. In the recent few years, an atypical influenza, avian flu, emerged. This infection brought a concern to the medical society. In early of this year, 2009, the newest emerging viral infection caused by a novel influenza virus, swine flu occurred and became pandemic. The topic on the new influenza virus becomes the present hot issue. Focusing on the contamination of classical influenza virus in semen, there are many evidences confirming the existence of virus in semen derived from the infected cases. It is also confirmed that the existence of the influenza virus in semen lead to decreased semen quality and pathological spermatozoa (6, 7). For the case of avian flu and swine flu, there is no report on the existence of virus in the semen of infected cases at present (8). However, a recent report on animal model indicated for the possibility of transmission of swine flu virus via reproductive tract insemination (9). It is suggested that new atypical influenza can result in poor semen quality and might lead to further infertility (8). However, there is no report on contamination of influenza virus in banked semen. A possible explanation might be the fact that although the influenza virus can contaminate in donated semen it leads to poor semen quality but no proof for possible further transmission to the other one. For the case of avian flu and swine flu, there is also no report on the contamination in banked semen. 
